# High Affinity Binding of Indium and Ruthenium Ions by Gastrins

**DOI:** 10.1371/journal.pone.0140126

**Published:** 2015-10-12

**Authors:** Graham S. Baldwin, Graham N. George, M. Jake Pushie

**Affiliations:** 1 The University of Melbourne Department of Surgery, Austin Health, Heidelberg, Victoria 3084, Australia; 2 Molecular and Environmental Science Research Group Department of Geological Sciences, 114 Science Place, University of Saskatchewan, Saskatoon, S7N 5E2, Canada; Martin-Luther-Universität Halle-Wittenberg, GERMANY

## Abstract

The peptide hormone gastrin binds two ferric ions with high affinity, and iron binding is essential for the biological activity of non-amidated forms of the hormone. Since gastrins act as growth factors in gastrointestinal cancers, and as peptides labelled with Ga and In isotopes are increasingly used for cancer diagnosis, the ability of gastrins to bind other metal ions was investigated systematically by absorption spectroscopy. The coordination structures of the complexes were characterized by extended X-ray absorption fine structure (EXAFS) spectroscopy. Changes in the absorption of gastrin in the presence of increasing concentrations of Ga^3+^ were fitted by a 2 site model with dissociation constants (K_d_) of 3.3 x 10^−7^ and 1.1 x 10^−6^ M. Although the absorption of gastrin did not change upon the addition of In^3+^ ions, the changes in absorbance on Fe^3+^ ion binding in the presence of indium ions were fitted by a 2 site model with K_d_ values for In^3+^ of 6.5 x 10^−15^ and 1.7 x 10^−7^ M. Similar results were obtained with Ru^3+^ ions, although the K_d_ values for Ru^3+^ of 2.6 x 10^−13^ and 1.2 x 10^−5^ M were slightly larger than observed for In^3+^. The structures determined by EXAFS all had metal:gastrin stoichiometries of 2:1 but, while the metal ions in the Fe, Ga and In complexes were bridged by a carboxylate and an oxygen with a metal-metal separation of 3.0–3.3 Å, the Ru complex clearly demonstrated a short range Ru—Ru separation, which was significantly shorter, at 2.4 Å, indicative of a metal-metal bond. We conclude that gastrin selectively binds two In^3+^ or Ru^3+^ ions, and that the affinity of the first site for In^3+^ or Ru^3+^ ions is higher than for ferric ions. Some of the metal ion-gastrin complexes may be useful for cancer diagnosis and therapy.

## Introduction

The peptide hormone gastrin (ZGPWLEEEEEAYGWMDFamide, Gamide) stimulates gastric acid secretion, and is an important growth factor for the gastric mucosa.[1] The biological effects of Gamide are mediated by the cholecystokinin2 receptor (CCK2R), which is a member of the G-protein-coupled receptor superfamily. Several different tumor types often express the CCK2R. In particular Reubi and coworkers have demonstrated that more than 90% of medullary thyroid carcinomas and ovarian stromal carcinomas, and more than 50% of astrocytomas and small cell lung carcinomas, are CCK2R-positive.[2] In contrast, non-amidated forms such as glycine-extended gastrin (Ggly), which are not recognised by the CCK2R, stimulate proliferation in the normal colorectal mucosa. Ggly and the gastrin precursor progastrin also accelerate the development of colorectal cancer.[3,4]

There has already been considerable interest in the use of metal chelate-conjugated gastrin derivatives for the diagnosis of CCK2R-positive tumors.[5] For example, the chelating group 1,4,7,10-tetraazacyclodecane-1,4,7,10-tetraacetic acid (DOTA) has been coupled to minigastrin_11_ (d-Glu-Ala-Tyr-Gly-Trp-Met-Asp-Phe-NH_2_) and radiolabelled with ^111^In or ^68^Ga.[6] One disadvantage of this approach is that incorporation of the metal ion requires harsh conditions (pH 4.5, 98°C, 15 min), which may result in some oxidative damage or modification to the peptide.[7]

Gastrins bind two ferric ions,[8] the first to Glu7 and the second to Glu8 and Glu9.[9] Ferric ions are essential for the biological activity of non-amidated forms of the peptide as a stimulant of cell proliferation and migration.[9] Thus, the biological activity of Ggly can be completely blocked, either by mutation of Glu7→Ala, or treatment with the iron chelator desferrioxamine. Bi^3+^ ions, on the other hand, by competing for the ferric ion binding site of Ggly, block biological activity *in vitro*[10] and in the normal colorectal mucosa in both mice and rats *in vivo*.[11] In contrast, ferric ions were not required for the biological activity of Gamide.[12] In the present study the binding of several other metal ions to Gamide and Ggly was investigated either via changes in absorption on addition of the metal ion itself, or by alterations in the absorption of the ferric ion-gastrin complex in the presence of the metal ion. The structures of several of the complexes were also characterized by extended X-ray absorption fine structure (EXAFS) spectroscopy. The metal ions structurally characterized in this study were chosen because each has radioisotopes which are advantageous for biomedical imaging (^67^Ga, ^68^Ga, ^97^Ru, ^109^In, and ^111^In) or radiotherapy (i.e. ^106^Ru), and which may be exploited in future studies.

## Materials and Methods

### Peptides and metal ions

Gamide and Ggly (88 and 93% pure, respectively) were purchased from Auspep (Clayton, Australia). The impurities consisted of water and salts. Solutions of metal ions (Aldrich, St. Louis, MO) were prepared in 10 mM HCl, and their concentrations determined by inductively coupled plasma-atomic emission spectroscopy at the National Measurement Institute (Pymble, Australia).

### Absorption spectroscopy

The 280 nm absorption of peptides (10 μM in 10 mM sodium acetate, pH 4.0, containing 100 mM NaCl and 0.005% Tween 20) in the presence of increasing concentrations of metal ions was measured against a buffer blank, in 1 ml quartz cuvettes thermostatted at 298 K, with a Cary 5 spectrophotometer (Varian, Mulgrave, Australia). This wavelength was chosen because the absorption maxima of the Ggly and Gamide peptides are at 280 nm, and because there is a peak in the difference absorption spectrum (Ggly–GglyFe) at the same wavelength.

### Curve fitting and statistics

Data (expressed as means ± S.E.M.) were fitted to one-site or two-site ordered models with the program BioEqs.[13,14] Because of the large number of parameters and the limited number of data points, the experimentally determined equilibrium constants and absorbance ratios given in Table 1 for the interaction of Gamide or Ggly with ferric ions were held constant while fitting the data for the interaction of other metal ions with Gamide or Ggly in the presence of ferric ions.

**Table 1:** Binding of metal ions by Gamide and Ggly. The affinity of, and the percentage absorbance change at 280 nm on, ferric or gallium ion binding to Gamide or Ggly were determined by fitting the mean data obtained in the absorbance experiments, described in the Fig 2 legend, to the models shown in Fig 1 with the program BioEqs. In the case of indium or ruthenium ions the corresponding values were obtained from fitting ferric ion titrations in the presence of various concentrations of indium or ruthenium ions, as described in the legends to Figs 3 and 4, respectively.

	Gamide							Ggly						
	K_d1_ (M)	A_280_ (%)	K_d2_ (M)	A_280_ (%)	K_d3_ (M)	A_280_ (%)	χ^2^	K_d1_ (M)	A_280_ (%)	K_d2_ (M)	A_280_ (%)	K_d3_ (M)	A_280_ (%)	χ^2^
Fe^3+^	3.0x10^-10^	100.0	8.5x10^-11^	313.4			5.5	5.7x10^-9^	100.0	7.0x10^-9^	365.9			5.7
Ga^3+^	3.3x10^-7^	100.0	1.1x10^-6^	335.8			4.1	1.7x10^-8^	100.0	2.3x10^-6^	340.2			14.4
In^3+^	6.5x10^-15^	100.0	1.7x10^-7^	74.1	4.0x10^-9^	217.7	3.3	2.1x10^-13^	100.0	1.4x10^-5^	72.8	9.6x10^-8^	255.6	19.4
Ru^3+^	2.6x10^-13^	100.0	1.2x10^-5^	178.6	1.7x10^-8^	264.9	26.1	5.3x10^-15^	100.0	3.6x10^-4^	1714.8	1.2x10^-6^	304.1	10.2

### X-ray absorption sample preparation, spectroscopy, and analysis

Samples for X-ray absorption spectroscopy (XAS) were prepared with 1 mM peptide, 50 mM MOPS, 10% DMSO and 20% glycerol as a cryoprotectant. Metal stock solutions were prepared from the corresponding nitrate salt, or Ru^III^Cl_3_ in the case of ruthenium, and titrated to a final concentration of 2 mM. Following data collection, samples containing 2 mM Ru or In were further titrated with 1 mM Fe for comparison. All samples were frozen in liquid N_2_ within 5 minutes of mixing, prior to data collection. XAS measurements were conducted at the Stanford Synchrotron Radiation Laboratory with the SPEAR storage ring containing roughly 450 mA at 3.0 GeV, using the data acquisition program XAS Collect.[15] Iron, gallium, indium, and ruthenium K-edge data were collected on the structural molecular biology XAS beamline 7–3, operating with a 20-pole 2 Tesla wiggler source, and employing a Si(220) double-crystal monochromator. For Fe and Ga spectroscopy a downstream vertically collimating Rh-coated mirror was employed for harmonic rejection, such that the harmonic fell above the cutoff. Incident X-ray intensity was monitored using a nitrogen-filled ionization chamber and X-ray absorption was measured as the X-ray Kα fluorescence excitation spectrum using an array of 30 germanium detectors (Canberra Industries, Meriden, CT, USA).[16] X-ray fluorescence was collected through a Soller slit assembly, and scattered X-rays were preferentially removed using filters of 6 or 9 absorption unit thickness (Mn for Fe, Zn for Ga, Ag for In and Mo for Ru) in order to maintain the count rates registered by the detector in the linear regime. During data collection, samples were maintained at a temperature of approximately 10 K using a liquid helium flow cryostat (Oxford Instruments, Abingdon, UK). For each data set, 6 scans for each sample were accumulated (14 scans for the Ru data), and the energy was calibrated by reference to the absorption of a reference foil of the same element, measured simultaneously with each scan (assuming a lowest energy inflection point of 7,111.3 eV for Fe, 10,368.2 eV for Ga, 27,940.0 eV for In and 22,118 eV for Ru). XAS data was collected using a multiple region strategy in order to optimize data processing and statistical significance across the numerous regions of the EXAFS experiment. Initially data was collected in 10 eV steps to provide an accurate measure of the background, then in 0.2 eV steps across the absorption edge (from 40 eV below the edge up to the threshold energy, *k* = 0 Å^-1^) for Fe and Ga, and 0.5 eV steps for the broader absorption edges of In and Ru. After the threshold energy, steps of 0.045 Å^-1^ were used for the remainder of the spectrum. Data collection used a count time of 3s per data point up to the threshold energy, then a count time weighted by *k* ^2^ was employed, ranging from 3s at the threshold energy up to a maximum of 12s at the end of the collected *k*-range. Average data collection time per scan was 40 minutes for each element.

The EXAFS oscillations χ(*k*) were quantitatively analyzed by curve-fitting using the EXAFSPAK suite of computer programs (http://www-ssrl.slac.stanford.edu/exafspak.html) as described by George *et al*.,[17] using *ab initio* theoretical phase and amplitude functions calculated with FEFF v8.20x5.[18] The energy thresholds of the EXAFS oscillations (*k* = 0 Å^-1^) were assumed to be 7,130 for Fe, 10,385 for Ga, 27,960 eV for In and 22,135 eV for Ru. Iron data was collected to a *k*-range of 14.2 Å^-1^, Ga to *k* of 14.0 Å^-1^, In to *k* of 16.2 Å^-1^ and Ru to *k* of 18 Å^-1^.

## Results

### Binding of ferric ions to gastrins

**Fig 1:**
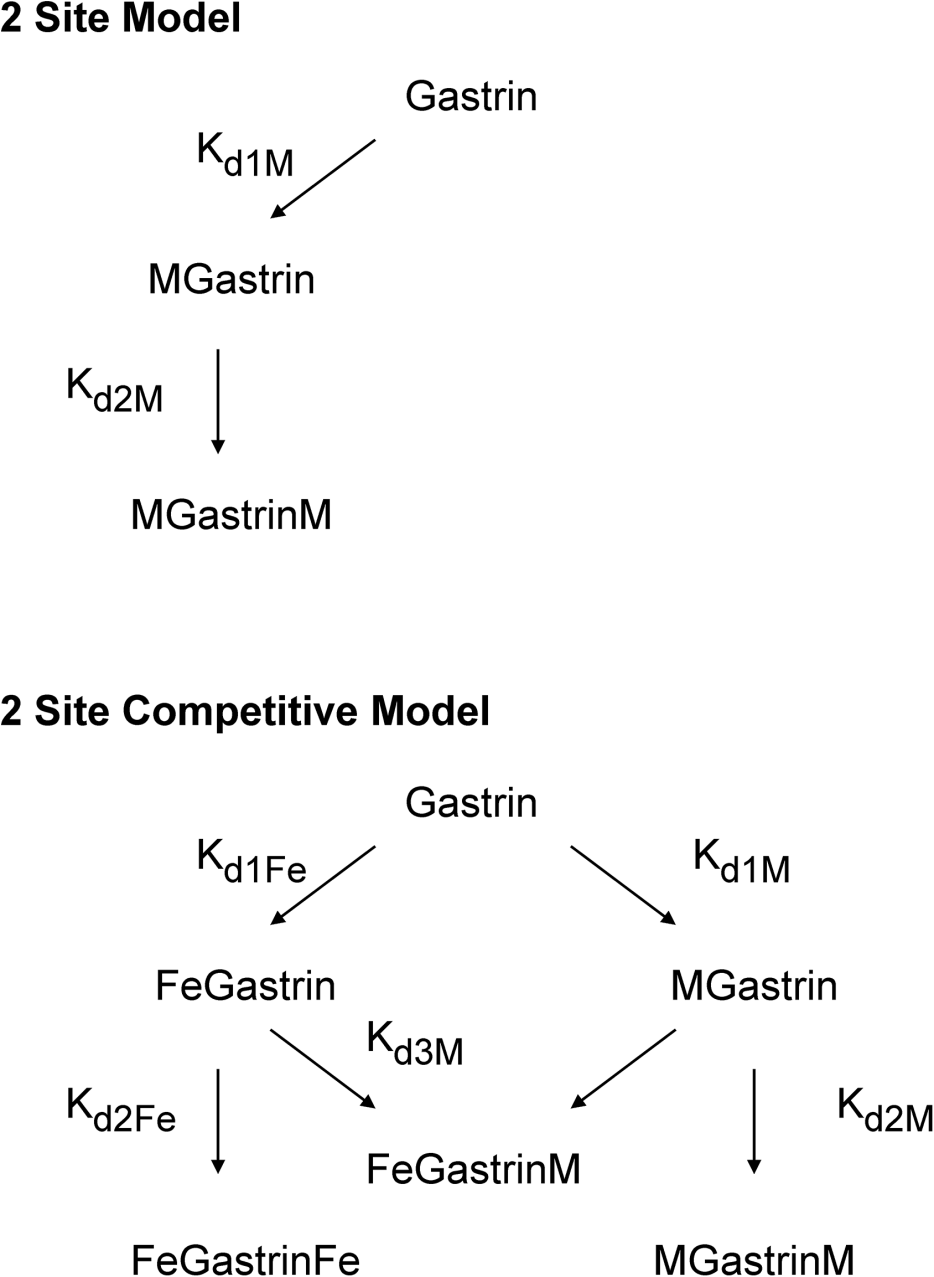
Models of metal ion binding. In the 2 site model gastrin binds two metal ions with dissociation constants K_d1M_ and K_d2M_. In the 2 site competitive model gastrin binds two ferric ions with dissociation constants K_d1Fe_ and K_d2Fe_, and two metal ions (M) to the same two sites with dissociation constants K_d1M_ and K_d2M_. The dissociation constant K_d3M_ describes the formation of the mixed FeGastrinM complex.

**Fig 2:**
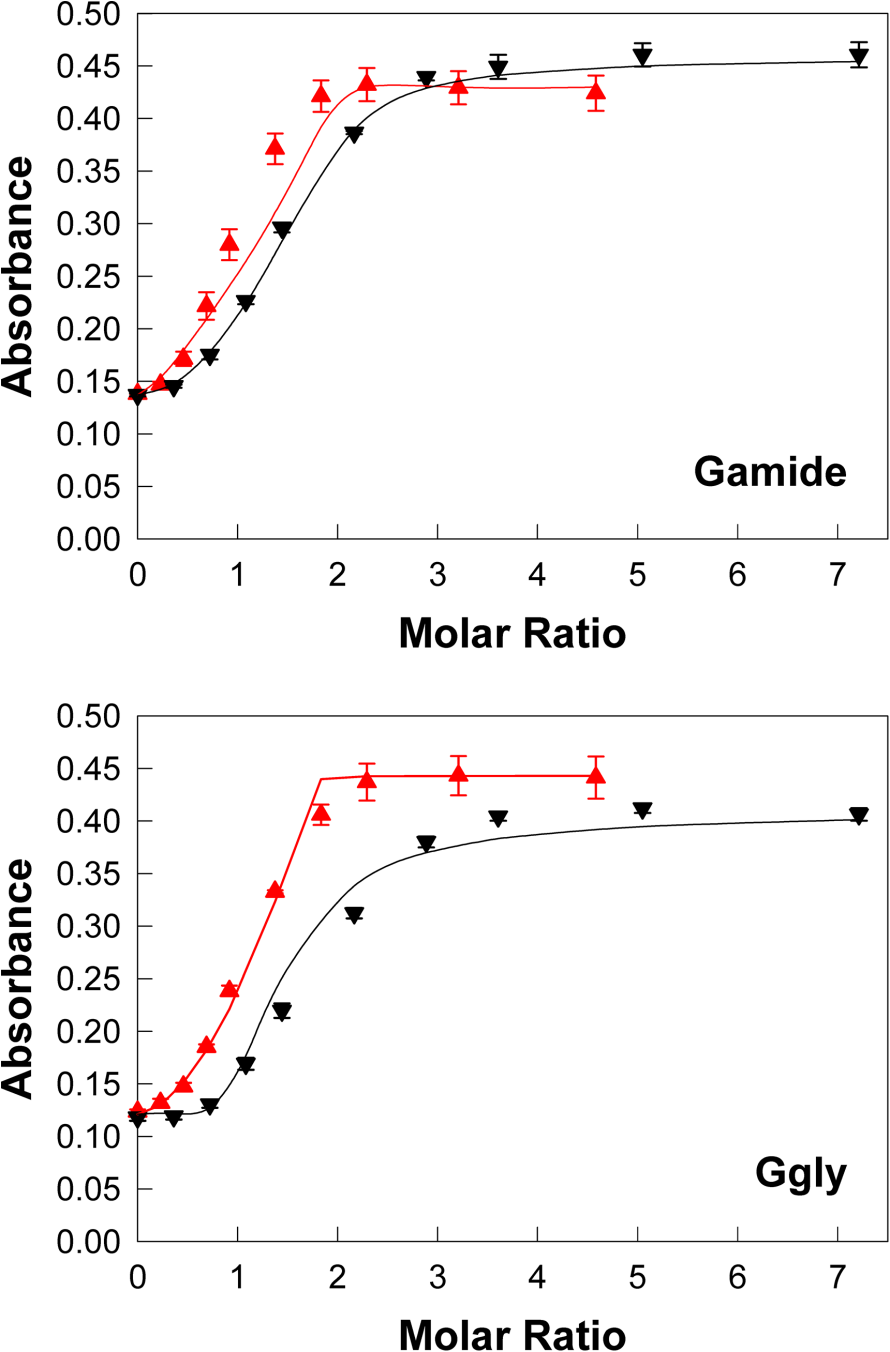
Ferric or gallium ions enhance gastrin absorbance. At pH 4.0 addition of aliquots of ferric chloride (red π) or gallium nitrate (black ▼) to 10 μM Gamide or Ggly in 10 mM Na^+^ acetate, 100 mM NaCl, 0.005% Tween 20 at 298 K resulted in an increase in the absorption at 280 nm. Points are means of at least three separate experiments; bars represent the SEM. Lines represent the best fit to the two site model shown in Fig 1 with the program BioEqs; the appropriate K_d_ and maximum absorbance values are given in Table 1.

The effect of addition of Fe^3+^ ions on the absorption spectrum and fluorescence of Gamide and Ggly at pH 4.0 has been reported previously.[8] The changes in absorption indicated a stoichiometry of binding of 2 mol Fe^3+^ /mol of peptide, and fitting of a linear transformation of the fluorescence data was consistent with 2 binding sites with μM affinities. Because of the lapse in time since the previous experiments, new spectra (S1 Fig) and absorption data sets were obtained, and fitted to the 2 site model shown in Fig 1 with the program Bioeqs as described in the Materials and Methods section. Reasonable fits were obtained with affinities of 3.0 x 10^−10^ and 8.5 x 10^−11^ M for Gamide and 5.7 x 10^−9^ and 7.0 x 10^−9^ M for Ggly (Fig 2, Table 1).

### Binding of gallium ions to gastrins

Similar to the results with Fe^3+^, the addition of Ga^3+^ ions also caused a general increase in absorbance in the visible region, and in the peak centred at 280 nm in the UV region of the spectrum (S1 Fig). Fitting of the increase in absorption at 280 nm for both Gamide and Ggly at pH 4.0 (Fig 2) with the program Bioeqs yielded affinities for Ga^3+^ of 3.3 x 10^−7^ and 1.1 x 10^−6^ M for Gamide and 1.7 x 10^−8^ and 2.3 x 10^−6^ M for Ggly (Table 1).

### Binding of indium and ruthenium ions to gastrins

**Fig 3:**
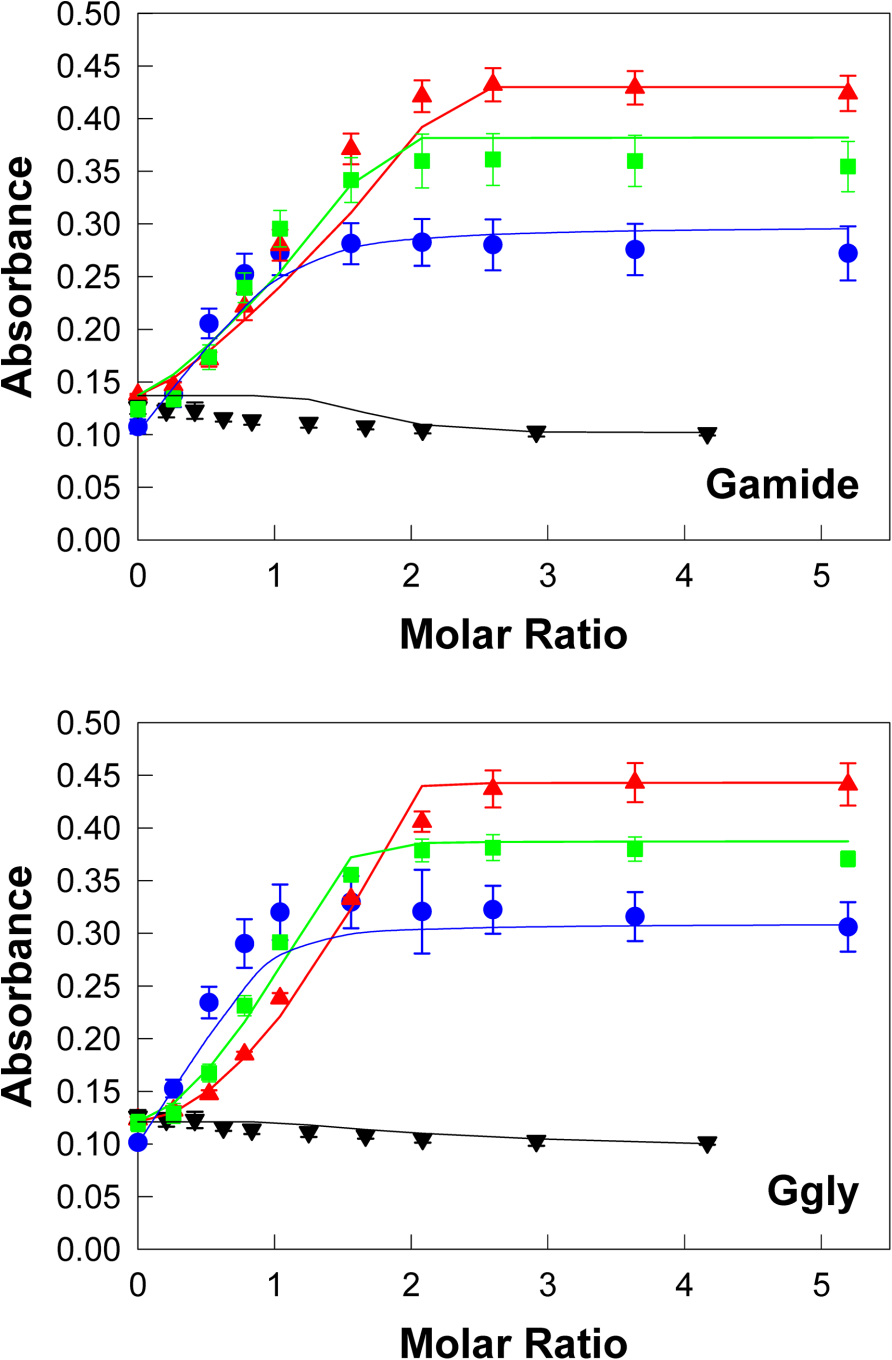
Indium ions compete with ferric ions for the gastrin binding sites. Addition of aliquots of indium nitrate (black ▼) to 10 μM Gamide or Ggly in the buffer described in the Fig 2 legend resulted in little change in absorbance at 280 nm when compared to the changes seen on addition of aliquots of ferric chloride (red π). However in the presence of 3.99 (green ⬛) or 39.85 μM (blue •) indium nitrate the changes in absorbance seen on addition of aliquots of ferric chloride were considerably different from the changes seen in the absence of indium nitrate. The points are means from three separate experiments; bars represent the SEM. The lines were constructed with the dissociation constants and maximum absorbance values (Table 1) obtained by fitting the data to the 2 site competitive model shown in Fig 1 with the program BioEqs.

The addition of In^3+^ ions caused little if any change in the absorption spectrum of Ggly at pH 4.0 (S1 Fig). However, in the presence of 39.85 μM In^3+^ ions, the absorbance at 280 nm for both Gamide and Ggly on addition of Fe^3+^ ions increased more rapidly and approximated to the curve expected for single site binding, with the maximum absorbance reached near a molar ratio of 1 (Fig 3). These observations suggest that an In^3+^ ion can bind to the first Fe^3+^ ion binding site with greater affinity than a Fe^3+^ ion, but without causing any change in absorbance. Indeed In^3+^ ions appear to compete for both Fe^3+^ ion binding sites, since the family of curves obtained at increasing concentrations of In^3+^ ions could be fitted with the program Bioeqs to the competitive two site model presented in Fig 1. The best fit affinities of ions for the first metal binding site were substantially higher than for Fe^3+^ ions, with K_d_ values for In^3+^ of 6.5 x 10^−15^ and 2.1 x 10^−13^ M for Gamide and Ggly, respectively (Table 1).

**Fig 4:**
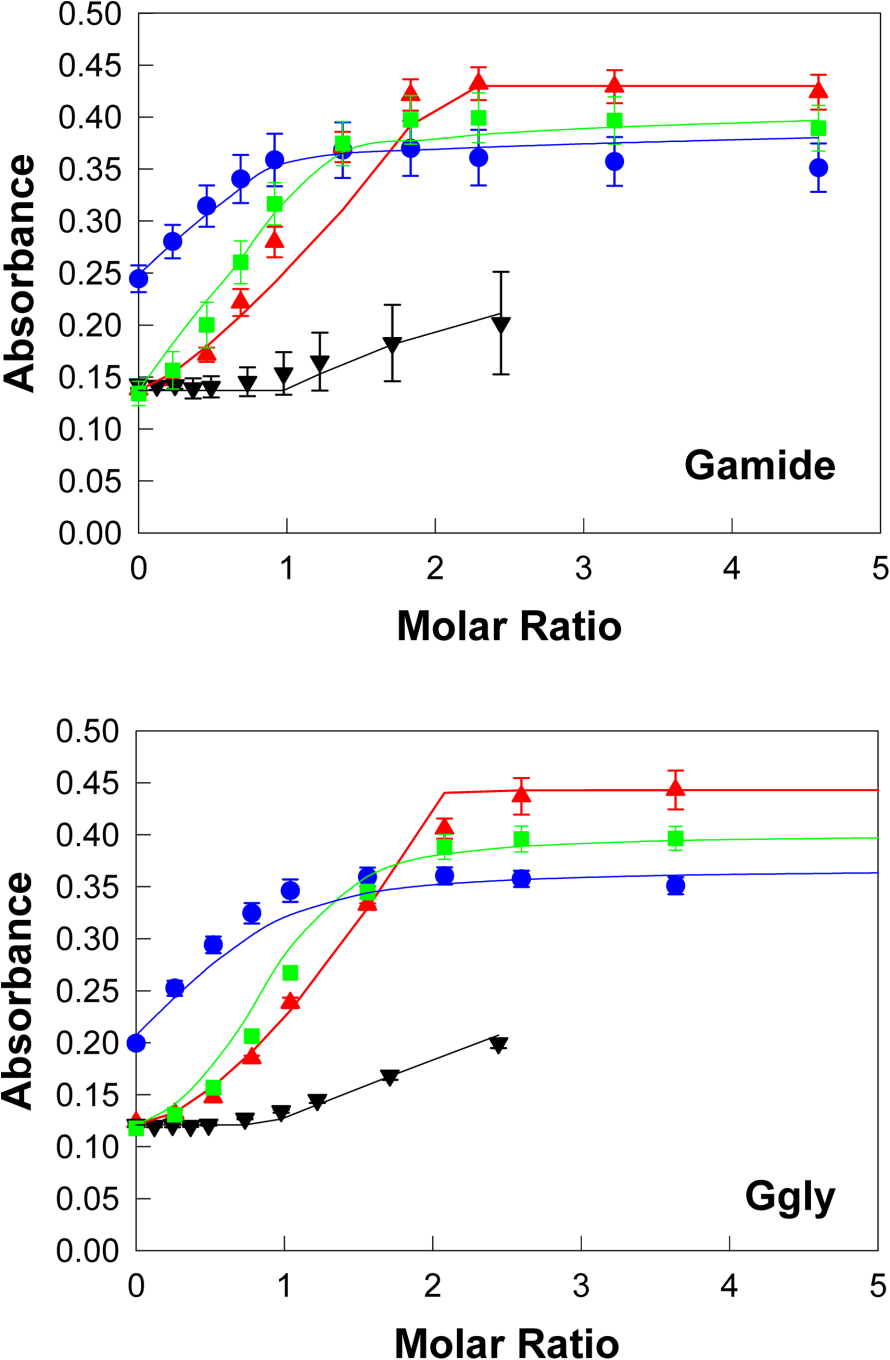
Ruthenium ions compete with ferric ions for the gastrin binding sites. Addition of aliquots of ruthenium chloride (black ▼) to 10 μM Gamide or Gly in the buffer described in the Fig 2 legend resulted in an increase in absorbance at 280 nm which was significantly less than the changes seen on addition of aliquots of ferric chloride (red π). However in the presence of 5.30 (green ⬛) or 26.48 μM (blue •) ruthenium chloride the changes in absorbance seen on addition of aliquots of ferric chloride were considerably different from the changes seen in the absence of ruthenium chloride. The points are means from three separate experiments; bars represent the SEM. The lines were constructed with the dissociation constants and maximum absorbance values (Table 1) obtained by fitting the data to the 2 site competitive model shown in Fig 1 with the program BioEqs.

Similar families of curves were obtained when the experiments were repeated with Ru^3+^ ions instead of In^3+^ ions (Fig 4). The major difference observed was that addition of Ru^3+^ ions itself caused a noticeable increase in absorbance at 280 nm for both Gamide and Ggly at pH 4.0 (S1 Fig). Nevertheless, the family of curves obtained at increasing concentrations of Ru^3+^ ions was reasonably well fitted with the program Bioeqs to the competitive two site model presented in Fig 1. The best fit affinities of Ru^3+^ ions for the first metal binding site were again substantially higher than for Fe^3+^ ions, with K_d_ values for Ru^3+^ of 2.6 x 10^−13^ and 5.3 x 10^−15^ M for Gamide and Ggly, respectively (Table 1).

### EXAFS characterization of Fe_2_Ggly

The XAS K-edge near-edge spectrum of Fe^III^_2_Ggly (S2 Fig) demonstrates pre-edge peaks centred at 7,114 eV arising from 1s → 3d(t_2g_) and 1s → 3d(e_g_) transitions (see inset plot). The relatively large separation between these peaks (Δ = 1.2 eV) arises from an elevation of the e_g_ levels, relative to the lower t_2g_ levels, and is indicative of low spin ferric iron. This large splitting also agrees with the expectation that the ferric ions are coordinated predominantly by hard ligands (i.e. the carboxylate donors of the Glu side chains). The observation that the near-edge spectrum does not display any apparent contributions from reduced ferrous forms of iron indicates that there was no appreciable photoreduction of the iron centres over the course of data collection.

**Fig 5:**
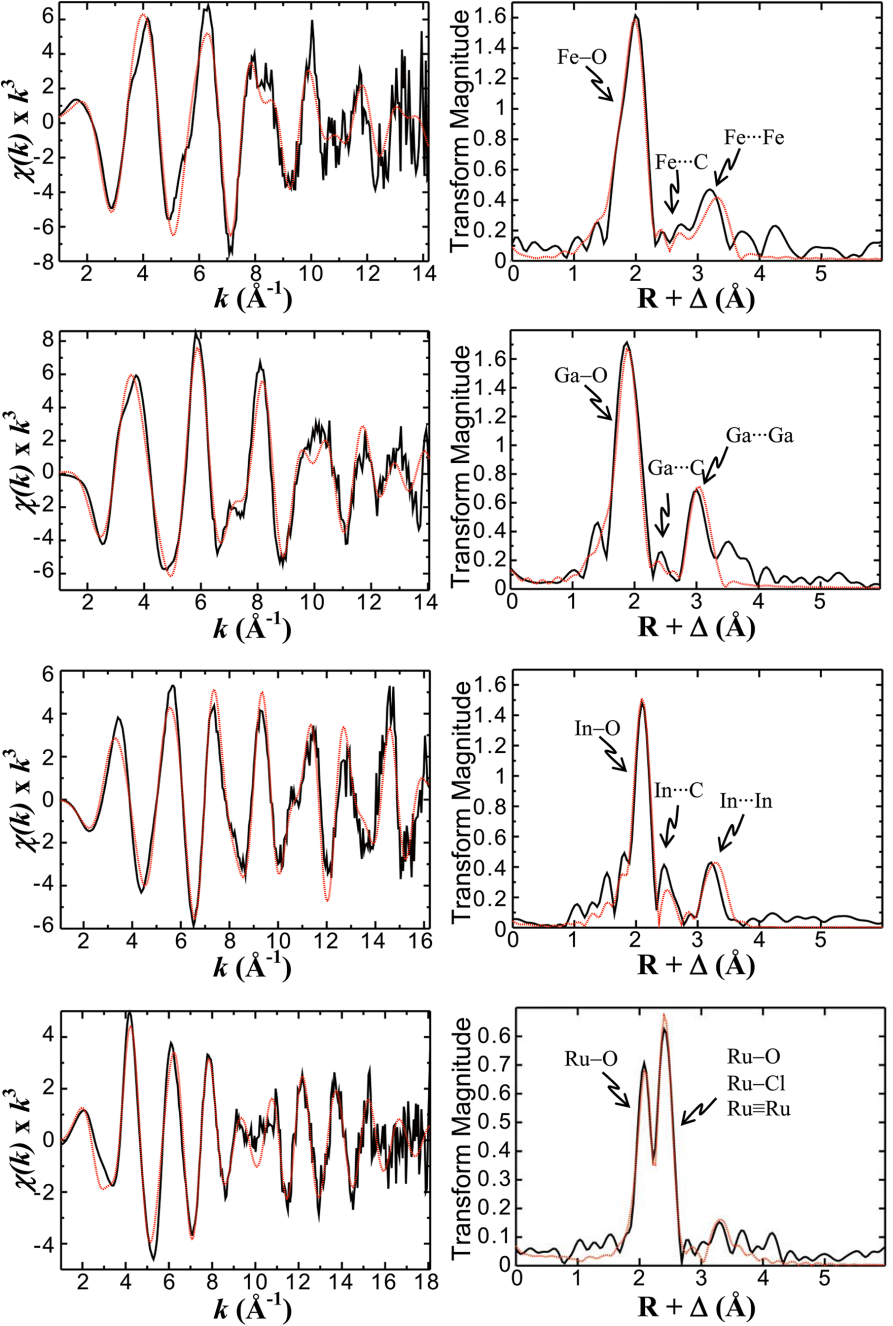
EXAFS spectra.

The Fe^III^_2_Ggly EXAFS data (Fig 5B) is dominated by Fe-O backscattering interactions just below 2 Å, and an outer shell backscattering Fe∙∙∙Fe interaction at ~3.3 Å (Fig 5B). The best fit to the data was obtained using single scattering paths, including 2 short Fe-O backscattering interactions at 1.90 Å, 4 Fe-O interactions at 2.03 Å, 1 Fe∙∙∙C interaction at 2.57 Å, 2 Fe∙∙∙C interactions at 2.96 Å and a single Fe∙∙∙Fe interaction at 3.33 Å (Table 2). The structural parameters are reminiscent of the diferric non-heme iron-binding proteins, such as methane monooxygenase and similar di-iron complexes, where the iron atoms are relatively close together and are bound by multiple carboxylates, including bridging carboxylates between the metal centres.[19–21] Based on the number of coordinating ligands and longer range Fe∙∙∙C scattering interactions, which appear prominent in the EXAFS data, the two ferric ions are predominantly bound by carboxylate donors with at least one bridging carboxylate. There is also a clear preference for inclusion of shorter Fe-O bond lengths (1.90 Å) in the fit, which may be indicative of bridging oxygen atoms, possibly as O^2-^ or OH^-^, although the internuclear separation is not particularly diagnostic in this case as mono-dentate carboxylate donors to Fe^3+^ can also fall close to this range of interatomic distances in similar complexes. In a search of the Cambridge Structural Database[22,23] for compatible candidate small molecule structures that are in agreement with the EXAFS fit parameters and that fulfill the requirement for coordination primarily by carboxylate donors, the best candidate structure identified (FEMTEX) contains a di-iron(II) site with bridging water molecules.[24] If one or two bridging oxygen atoms derived from water are present in the Fe^III^_2_Ggly coordination environment they are more likely to exist in a deprotonated state, such as OH^-^, which would provide sufficient charge compensation with 4–5 carboxylate donors to make the overall charge zero or minus 1.

**Table 2:** EXAFS curve fitting results.^a^

	*Path*	*N*	*R*	*σ* ^*2*^	*ΔE*_*0*_	*F*
Fe^III^_2_Ggly						
	Fe-O	2	1.902(6)	0.0025	- 6.7(6)	0.4129
	Fe-O	4	2.029(4)	0.0025		
	Fe…C	1	2.57(2)	0.0045		
	Fe…C	2	2.96(2)	0.0045		
	Fe…Fe	1	3.330(6)	0.0035		
Ga^III^_2_Ggly						
	Ga-O	2	1.877(4)	0.0025	- 7.8(6)	0.3143
	Ga-O	3	1.985(3)	0.0025		
	Ga…C	1	2.60(2)	0.0045		
	Ga…C	2	3.02(1)	0.0045		
	Ga…Ga	1	3.046(4)	0.0035		
In^III^_2_Ggly						
	In-O	1	1.979(6)	0.0025	- 14.6(5)	0.3217
	In-O	5	2.132(2)	0.00225		
	In…C	2	2.635(7)	0.0045		
	In…C	2	3.09(2)	0.0045		
	In…In	1	3.266(3)	0.0035		
Ru^III^_2_Ggly						
	Ru-O	2	2.069(4)	0.0032	+ 3.6(5)	0.3884
	Ru-O	2	2.173(6)	0.0032		
	Ru-Cl	0.5	2.51(1)	0.0033		
	Ru≡Ru	1	2.418(4)	0.0040		
	Ru-O	0.5	2.42(5)	0.0053		
	Ru…O…Ru	2	3.30(1)	0.0054		

a Coordination numbers, *N*, interatomic distances *R* (Å), Debye-Waller factors *σ* ^*2*^ (Å^2^), and threshold energy shift Δ*E*_*0*_ (eV), were derived from EXAFS curve-fitting. The fit error parameter *F* is defined as $F=\sqrt{\sum k^{6}{\left(\chi {\left(k\right)}_{calc}-\chi {\left(k\right)}_{expt}\right)}^{2}/\sum k^{6}\chi {\left(k\right)}_{expt}^{2}}$, with the summation being over data points included in the fit. Values in parentheses are the estimated standard deviations obtained from the diagonal elements of the covariance matrix; these are precisions and are distinct from the accuracies which are expected to be larger (*ca* ± 0.02 Å for *R*, and ± 20% for *N* and *σ* ^*2*^), although relative accuracies (e.g. comparing two different Fe—O bond-lengths) will be more similar to the precisions.

The K-edge EXAFS spectra (A, C, E, G, solid black lines) and their corresponding Fourier transforms (B, D, F, H) for the complexes of Ggly with Fe^3+^ ions (A, B), Ga^3+^ ions (C, D), In^3+^ ions (E, F), or Ru^3+^ ions (G, H) are shown together with the best fits (red dashed lines) calculated using the single scattering path parameters listed in Table 2.

The EXAFS data was best fit by a single Fe∙∙∙Fe scattering interaction. This observation indicates that Ggly binds Fe^3+^ in a di-iron coordination environment, without apparent recruitment of any additional ferric ions, as is otherwise often encountered in multinuclear small molecule crystal structures of iron-carboxylate complexes. A best fit could also be obtained by including one or two short Fe-O scattering interactions at ~1.9 Å, which may be attributable to one or more bridging oxygen atoms, although this relatively short separation is also compatible with coordination by a bridging carboxylate. The Fe∙∙∙C scattering interactions suggest that each iron centre interacts with one to two bridging carboxylates as well as at least one additional carboxylate that is not involved in a bridging interaction.

### EXAFS characterization of Ga_2_Ggly

Although the primary backscattering peak in the Ga^III^_2_Ggly EXAFS Fourier transform (Fig 5D) appears more symmetric than in the analogous Fe^III^_2_Ggly complex, significantly improved fits were obtained with the inclusion of two separate Ga-O backscattering interactions: two at 1.88 Å and three at 1.99 Å. The fact that inclusion of a third Ga-O backscatterer at 1.88 Å did not significantly change the fit suggested that the Ga^3+^ centres could be either 5- or 6-coordinate, although mixtures cannot be ruled out either. The EXAFS data also clearly demonstrate a Ga∙∙∙Ga backscattering interaction at 3.05 Å, and the results from the single scattering path model used for the Ga^III^_2_Ggly data (Table 2) are in agreement with the di-iron EXAFS model, albeit with shorter internuclear separations overall. The structural implication is that Ga^3+^, when coordinating to Ggly, appears to substitute for Fe^3+^ with minimal structural change in the local coordination environment of the di-nuclear coordination site.

### EXAFS characterization of In_2_Ggly

The EXAFS Fourier transform (Fig 5F) for In^III^_2_Ggly shows a shoulder on the shorter distance side of the primary backscattering peak centred at 2.1 Å, and the inclusion of a short In-O backscattering interaction significantly improved fitting of the data. Truncating the *k*-range of the EXAFS data (Fig 5E) to 14 Å^-1^ confirmed that this apparent peak in the Fourier transform was reasonably well represented in the low *k*-range data, as would otherwise be expected for backscattering interactions with light atoms, such as oxygen, and was not attributable to noise or other artifacts. Overall the best fit to the EXAFS data was obtained by including a single short metal-O atom path at 1.98 Å as well as five equivalent In-O backscattering interactions at 2.13 Å. The In∙∙∙In backscattering interaction was observed at 3.26 Å, and such short internuclear separations have been previously reported for In complexes with bridging light atoms.[25] The fact that the fit parameters for In^III^_2_Ggly agreed reasonably well with those used for the parent Fe^III^_2_Ggly complex suggested that, like Ga^3+^, In^3+^ coordinates to Ggly within a di-indium binding environment similar in structure to the Fe^3+^ complex.

### EXAFS characterization of Ru_2_Ggly

The EXAFS Fourier transform of the di-Ru^3+^ complex (Fig 5H) is significantly different from those of the other complexes investigated and displays two intense primary backscattering peaks centred at ~2.1 Å and ~2.4 Å. The magnitude of the Fourier transform peaks in Fig 5H is greatly diminished compared to those of the other complexes shown in Fig 5B, 5D and 5F and is the result of significant cancellation between individual Ru scattering paths. The best fit to the data was obtained using a dinuclear Ru^3+^ complex, containing a Ru–Ru core, a bridging carboxylate and the remaining coordination completed with O-atoms and a single chloride bound to one of the Ru centres. While the bond-length of the Ru-Ru coordination is similar to that observed for other backscatterers, such as Ru-Cl, confusion of the EXAFS with these alternatives is not possible because the Ru–Ru and Ru–Cl EXAFS differ in phase by approximately 180 degrees. Because the EXAFS experiment gives the superposition of all coordination environments about the Ru centres simultaneously, the fit parameters (Table 2) required fractional occupancy of Cl as well as fractional occupancy of an O-atom at ~2.4 Å in order to represent the contributions from the two non-equivalent Ru coordination environments. This mixed dinuclear coordination environment also gave the maximal EXAFS cancellation represented by the experimental data. The short internuclear separation (2.4 Å) between the Ru centres is indicative of a direct metal-metal bond.[26]

## Discussion

We have previously reported that gastrins bind two ferric ions with affinities in the μM range.[8,9] The discrepancy between these values and the values reported in Table 1 is probably due to the fact that the previous estimates were obtained by least squares fitting of a linear transformation of the fluorescence data, assuming binding sites with identical affinity. The present values were obtained by fitting the untransformed absorption data with the program BioEqs, which makes no such assumptions. We also reported previously that bismuth ions inhibit ferric ion binding, and analysis of the binding data with the program BioEqs was consistent with mixed inhibition, in which the gastrin-bismuth complex was still able to bind two ferric ions.[11] In the present study the binding of a range of other trivalent metal ions to gastrins was investigated by ultraviolet absorption spectroscopy. Both Gamide and Ggly bound Ga^3+^, In^3+^ or Ru^3+^; no binding of other trivalent metal ions from group 8/9 (Os^3+^, Rh^3+^), group 13 (Al^3+^, Tl^3+^), or group 15 (As^3+^, Sb^3+^) was detected (data not shown).

The binding of Ru^3+^ ions to gastrins was not unexpected, as ruthenium is in the same column of the periodic table as iron and therefore shares some chemical similarity. Analysis of the binding data (Fig 4) was consistent with competitive inhibition, in which ruthenium and ferric ions competed for the two metal ion binding sites on gastrin. The dissociation constants (Table 1) indicated that the affinity of gastrins for ruthenium ions was substantially higher than for ferric ions. In fact, the curve for ferric ion binding in the presence of 26.5 μM Ru^3+^ ions indicated that both Gamide and Ggly were able to bind only one Fe^3+^ ion under these conditions.

Some group 13 ions also bound to gastrins, likely due to their similar charge and atomic radius. Binding of Ga^3+^ ions resulted in an increase in the absorption of both Gamide and Ggly (Fig 2), and analysis of the binding data was consistent with the binding of two Ga^3+^ ions, with affinities substantially weaker than for ferric ions (Table 1). Addition of In^3+^ ions did not change the absorption of either Gamide or Ggly (Fig 3), but did modify the changes in the absorption of both Gamide and Ggly on subsequent addition of Fe^3+^ ions. The binding data for In^3+^ ions was reasonably well fitted by a competitive inhibition model, in which the indium and ferric ions competed for the two metal ion binding sites on gastrin. The dissociation constants (Table 1) indicated that the affinity of gastrins for indium ions was substantially higher than for ferric ions and similar to the values for ruthenium ions. Binding of the other group 13 ions, aluminium or thallium (Al^3+^, Tl^3+^), by gastrins was not detected by absorption spectroscopy (data not shown).

Evidence for the binding of the group 15 ion bismuth to gastrins has been presented previously.[10,11] The fact that the binding data was better fitted by a mixed inhibition model than a competitive model suggested that the gastrin-bismuth complex was still able to bind two ferric ions, and thus that the binding sites for the first bismuth and ferric ions were subtly different. Binding of the other group 15 ions, arsenic or antimony (As^3+^, Sb^3+^), by gastrins was not detected by absorption spectroscopy (data not shown). In previous studies no evidence was obtained for high affinity binding of a wide range of divalent metal ions to either Ggly[8] or the gastrin precursor, progastrin.[27] Hence the metal binding sites of gastrins appear thus far to be selective for trivalent metal ions of groups 8, 13 and 15.

The selectivity of the metal binding sites of transferrin has been investigated previously. Transferrin binds two ferric ions with high affinity, with bicarbonate-independent logK_1_ and logK_2_ values of 21.4 and 20.3, respectively.[28] Spectroscopic evidence has also been presented for the formation of complexes of transferrin with divalent (copper, nickel, zinc, etc.) and trivalent (aluminium, gallium, indium, etc.) metal ions (see review by Harris[29]). Although the variation in experimental conditions often renders comparisons of the data obtained by different groups invalid, the data of Harris and coworkers indicates that the order of decreasing affinity for logK_1_ is: iron, 21.4 > gallium, 19.8 > indium, 18.3 > aluminium, 13.7.[28] In every case the value is considerably higher than the corresponding affinity for gastrin, so that the mechanism previously proposed for catalysis of iron loading of apo-transferrin by gastrin[30] may also be valid for the other metal ions studied herein.

**Fig 6:**
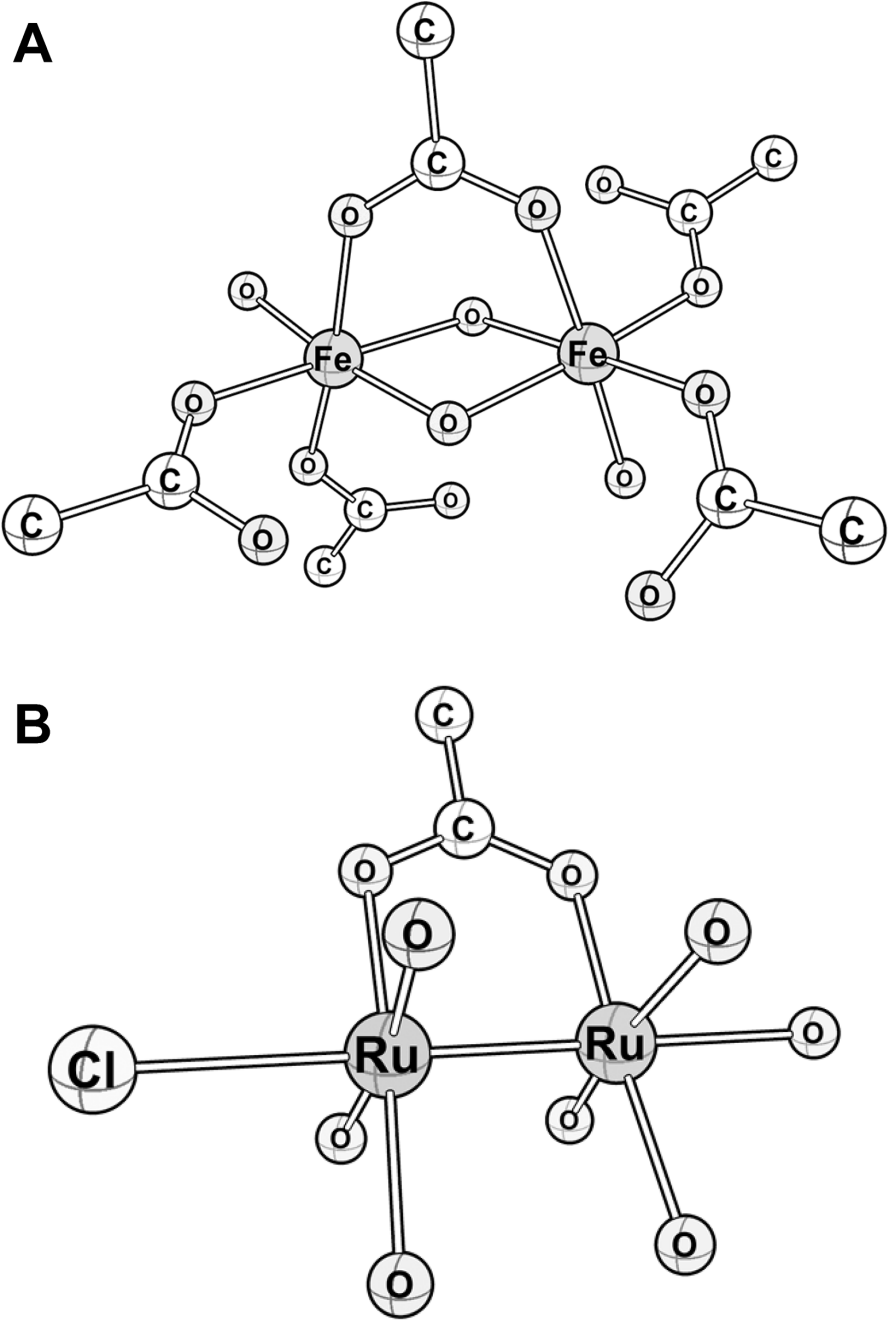
Proposed structural models of FeIII2Ggly and RuIII2Ggly. The model for Fe^III^_2_Ggly (A) is based on the EXAFS data presented in Fig 5B, and is consistent with previous NMR and visible spectroscopic studies of Ggly and mutant peptides.[8,9,12] The two Fe^III^ ions are coordinated by the carboxylate side chains likely from glutamates 6, 7, 8, 9 and 10, with glutamate 7 acting as a ligand to both Fe^III^ ions. One or more oxygens also act as bridging ligands between the two Fe^III^ ions. The peptide backbone and non-coordinating side chains have been omitted for simplicity. The model for Ru^III^_2_Ggly (B) is based on the EXAFS data presented in Fig 5H, and differs from the model for Fe^III^_2_Ggly in the presence of a Ru≡Ru bond and a chloride ion ligand.

The structures of the complexes of glycine-extended gastrin_17_ with trivalent metal ions have been determined by EXAFS spectroscopy. Although the best fit Fe^3+^ and Ga^3+^ structures included shorter metal-O-atom donors (1.92 and 1.88 Å, respectively), these shorter distances are not necessarily indicative of bridging ligands and the structural parameters from the fit procedure, including the In^3+^ data, are also compatible with coordination solely by carboxylate donor ligands. Further evidence for this structure is provided by the preference for inclusion of longer-range metal∙∙∙C scattering interactions, corresponding to the central carbon atom of a coordinating carboxylate. Construction of a hypothetical model of the di-ferric form of Ggly, guided by parameters from the EXAFS curve fitting which requires 6-coordinate iron centres with at least one bridging carboxylate, is shown in Fig 6. Although the structural model includes recruitment of all five glutamates from the polyglutamate sequence in order to preserve the symmetry of the fit parameters between iron centres, the precise number of coordinating glutamates cannot be determined by EXAFS spectroscopic methods alone. Recruitment of glutamates in the coordination of Fe^3+^ to Ggly has been examined previously by ^1^H NMR and, although the resonances from Glu 7, 8 and 9 were the most significantly attenuated upon coordination of two Fe^3+^ ions, the amide protons of the other two glutamates were also affected.[9] The remainder of the coordinating light atoms at each iron centre in Fig 6 may be comprised of water-derived ligands and possibly amide carbonyls from the peptide backbone.

Surprisingly, the structure of the Ru^3+^-glycine-extended gastrin_17_ complex differed from the complexes with Fe^3+^, Ga^3+^ and In^3+^. A Ru≡Ru bond was observed, whereas in the other three structures the metal ions were separated by bridging oxygen atoms (Fig 6). A search of the Cambridge Structural Database for Ru complexes containing primarily O-atom donors and a Ru–Cl bond demonstrates a preponderance of dinuclear Ru≡Ru complexes, which are typically bridged by carboxylate-type donor ligands. Although only one bridging carboxylate donor was used for EXAFS curve fitting the remaining O-atom donors of the Ru_2_^6+^ core are also likely comprised of anionic carboxylate donors from the polyglutamate region of the peptide, as opposed to coordination by water or DMSO, in order to balance the charge of the cationic centre. The fact that an EXAFS investigation of di-ruthenium complexes containing bridging oxygen ligands (but using bipyridine to coordinate Ru) reported significantly different EXAFS spectra from those herein[31] further supports the conclusion that bridging O-atoms are not present in Ru^III^_2_Ggly.

Gastrin derivatives conjugated to metal chelates such as DOTA and radiolabelled with ^111^In or ^68^Ga have already been used for the diagnosis of CCK2R-positive tumors.[5,6] The data presented herein suggest that radioactive isotopes of Ga, Ru or In could be directly complexed with amidated gastrin_17_ itself for use as CCK2R probes in single-photon emission computed tomography (SPECT, ^67^Ga, ^97^Ru, ^111^In) and positron emission tomography (PET, ^68^Ga, ^109^In). The recent development of a portable generator for ^68^Ga makes the latter approach more feasible than previously.[32] One advantage of this approach would be that oxidative damage to the peptide[7] would also be avoided, since complex formation proceeds rapidly at room temperature.

In contrast to the abundant structure–function information available for the CCK2R, the identities of the receptors for non-amidated gastrins such as progastrin and Ggly are still controversial. The CCK2R does not bind either recombinant human progastrin[33] or synthetic Ggly[34], and the failure of CCK1R and CCK2R antagonists to inhibit binding of ^125^I-Ggly to the rat pancreatic cell line AR4-2J clearly differentiated the Ggly binding site from either of the known receptors.[34] A recent report has identified the F1-ATPase as a candidate Ggly receptor.[35] The identification of annexin II as the progastrin receptor[36], however, has been disputed.[37] The availability of novel Ggly derivatives radioactively labelled with In and Ru isotopes may assist in resolving the current controversy over the identity of the receptors for non-amidated gastrins. Since progastrin and Ggly stimulate proliferation in the normal colorectal mucosa and accelerate the development of colorectal cancer[3,4], identification of such receptors may lead to improvements in cancer diagnosis and therapy.

## Supporting Information

S1 Fig Absorption spectra of Ggly with and without metal ions. The full UV-visible spectra of Ggly (9.42 μM) in the absence of added metal ions (solid black lines), or in the presence of approximately 1 (dashed and dotted blue lines) or 2 (dashed red lines) mol/mol Fe^3+^, Ga^3+^, In^3+^, or Ru^3+^ ions, are shown. As reported previously,[8] the UV-visible spectrum of the Ggly-Fe complex is characterised by a peak centred on 280 nm, and a general increase in absorption throughout the visible range. The full UV-visible spectra of the 1:2 Ggly-Ga and 1:1 and 1:2 Ggly-Ru complexes are similar in shape, although the magnitude of the 280 nm peak differs in each case. No change in absorption was seen on addition of 1 mol/mol Ga^3+^ ions, or on the addition of 1 or 2 mol/mol In^3+^ ions. The exact molar ratios were: Fe, 0.98, 1.95; Ga, 0.95, 1.91: In, 0.97, 1.95; Ru, 0.97, 1.94. Data are the average of 3 separate experiments.
(TIF)

S2 Fig Fe K-edge near edge spectrum of FeIII2Ggly. The XAS K-edge near edge spectrum of Fe^III^_2_Ggly was collected as described in Materials and Methods. The pre-edge peaks centred at 7,114 eV (see inset) arise from 1s → 3d(t2_g_) and 1s → 3d(e_g_) transitions. The relatively large separation between these peaks (Δ = 1.2 eV) results from an elevation of the e_g_ levels, relative to the lower t2_g_ levels, and is indicative of low spin ferric iron in an octahedral-type coordination environment.
(TIF)

## Abbreviations

CCK2R: cholecystokinin2 receptor
DOTA: 1,4,7,10-tetraazacyclodecane-1,4,7,10-tetraacetic acid
Gamide: amidated gastrin_17_
Ggly: glycine-extended gastrin_17_
K_d_: dissociation constant
PET: positron emission tomography
SPECT: single-photon emission computed tomography
EXAFS: extended X-ray absorption fine structure
XAS: X-ray absorption spectroscopy
