# Supplementary figures and images for: High Affinity Binding of Indium and Ruthenium Ions by Gastrins
Source: PLoS One. 2015 Oct 12;10(10):e0140126. doi: 10.1371/journal.pone.0140126 (PMC4601796; doi:10.1371/journal.pone.0140126)

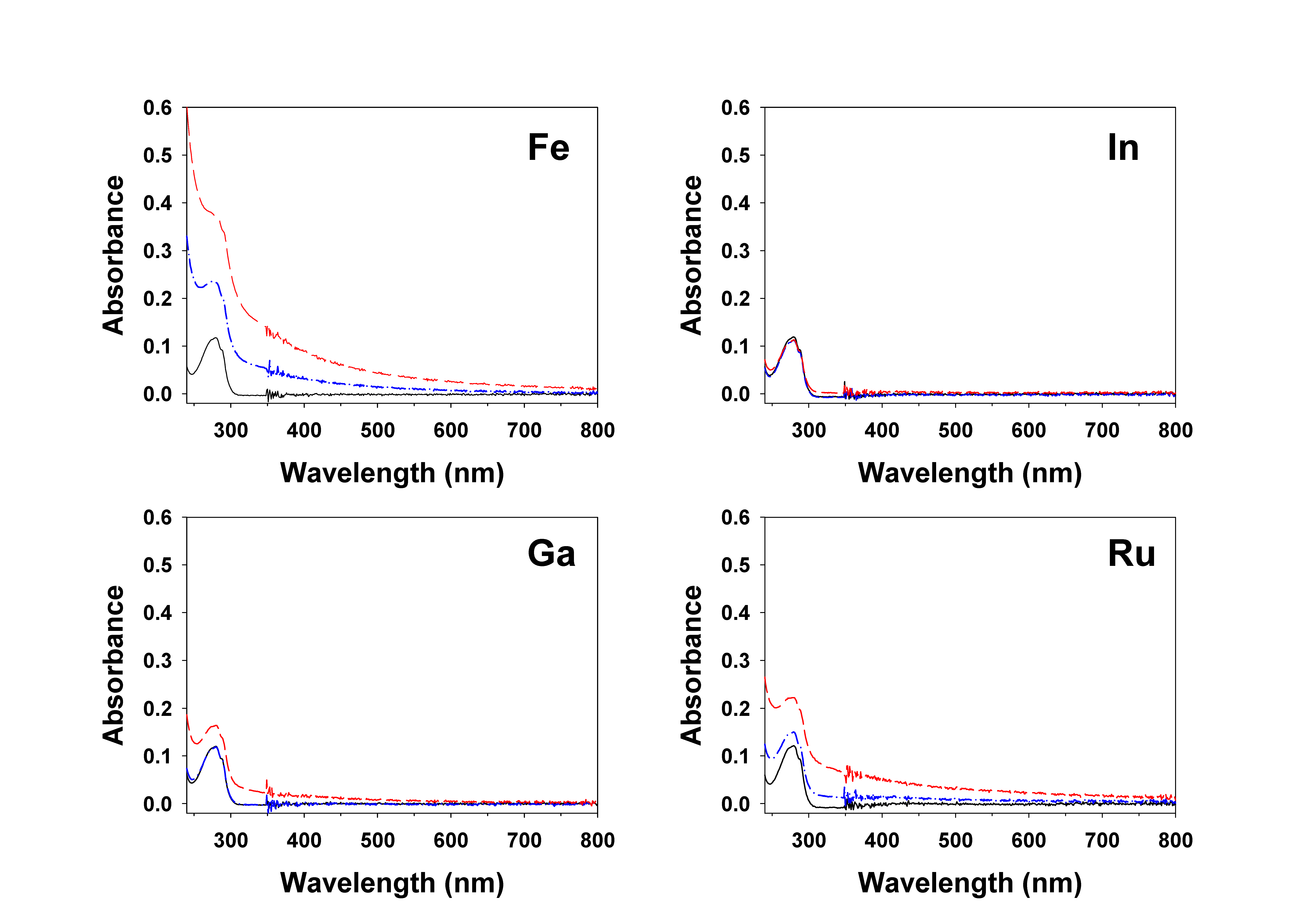

Supplement: S1 Fig — The full UV-visible spectra of Ggly (9.42 μM) in the absence of added metal ions (solid black lines), or in the presence of approximately 1 (dashed and dotted blue lines) or 2 (dashed red lines) mol/mol Fe3+, Ga3+, In3+, or Ru3+ ions, are shown. As reported previously,[8] the UV-visible spectrum of the Ggly-Fe complex is characterised by a peak centred on 280 nm, and a general increase in absorption throughout the visible range. The full UV-visible spectra of the 1:2 Ggly-Ga and 1:1 and 1:2 Ggly-Ru complexes are similar in shape, although the magnitude of the 280 nm peak differs in each case. No change in absorption was seen on addition of 1 mol/mol Ga3+ ions, or on the addition of 1 or 2 mol/mol In3+ ions. The exact molar ratios were: Fe, 0.98, 1.95; Ga, 0.95, 1.91: In, 0.97, 1.95; Ru, 0.97, 1.94. Data are the average of 3 separate experiments. (TIF) [file pone.0140126.s001.tif]

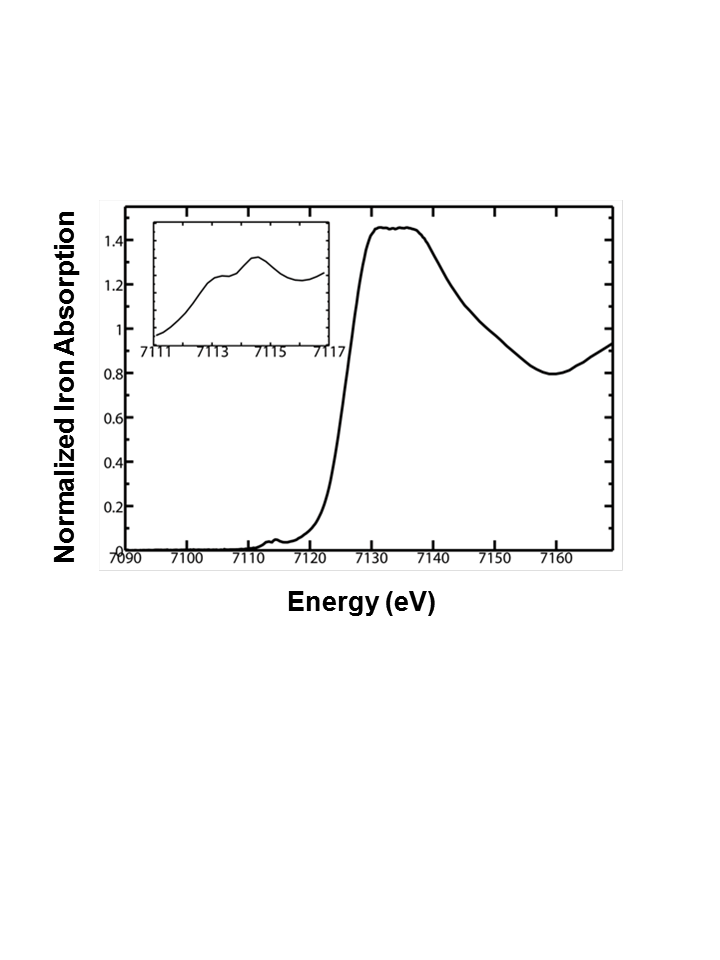

Supplement: S2 Fig — The XAS K-edge near edge spectrum of FeIII 2Ggly was collected as described in Materials and Methods. The pre-edge peaks centred at 7,114 eV (see inset) arise from 1s → 3d(t2g) and 1s → 3d(eg) transitions. The relatively large separation between these peaks (Δ = 1.2 eV) results from an elevation of the eg levels, relative to the lower t2g levels, and is indicative of low spin ferric iron in an octahedral-type coordination environment. (TIF) [file pone.0140126.s002.tif]
